# Disentangling the personality pathways to well-being

**DOI:** 10.1038/s41598-023-29642-5

**Published:** 2023-02-27

**Authors:** Paulo A. S. Moreira, Richard A. Inman, C. Robert Cloninger

**Affiliations:** 1 Instituto de Psicologia E de Ciências da Educação (IPCE), Universidade Lusíada Porto, Porto, Portugal; 2 Centro de Investigação Em Psicologia Para O Desenvolvimento (CIPD), Lisbon, Portugal; 3 Department of Psychiatry, Washington University in St. Louis, St. Louis, MO USA; 4 Anthropedia Foundation, St. Louis, USA; 5 Departamento de Educação e Psicologia, Faculdade de Ciências Humanas e Sociais, University of Trás-Os-Montes and Alto Douro (UTAD), Quinta de Prados, 5000-801 Vila Real, Portugal

**Keywords:** Psychology, Human behaviour

## Abstract

Recent genomic, psychological, and developmental research shows that human personality is organized as a complex hierarchy that ascends from individual traits in many specific situations to multi-trait profiles in two domains that regulate emotional reactivity (temperament) or goals and values (character), and finally to three integrated temperament-character networks that regulate learning to maintain well-being in changing conditions. We carried out person-centered analyses of the components of subjective well-being (positive affect, negative affect, and life satisfaction) to personality in both adolescents (N = 1739) and adults (N = 897). Personality was considered at each level of its organization (trait, temperament or character profiles, and joint temperament-character networks). We show for the first time that negative affect and life satisfaction are dependent on the personality network for intentional self-control, whereas positive affect is dependent on the personality network for self-awareness that underlies the human capacities for healthy longevity, creativity, and prosocial values.

## Introduction

A long tradition of research has shown that people tend to lead longer and healthier lives, and behave more prosocially, when they subjectively experience their lives positively rather than negatively^1,2^. Such subjective experiences, referred to as subjective well-being (henceforth SWB), capture the cognitive and emotional aspects of the subjective feelings a person has about their own life circumstances^3–5^. SWB is said to be higher when a person experiences a high level of positive emotions alongside a low level of negative emotionality, and when they also evaluate their life circumstances to be satisfactory according to relevant criteria and standards (e.g., values, goals, norms, and cultural variables). Thus, SWB is widely considered to have a tripartite structure comprising positive affect, negative affect, and life satisfaction. Findings from various empirical studies have suggested these three primary components are dissociable (e.g., low negative affect does not assure high positive affect), and so ought to be assessed and studied independently^6–8^. However, studies have also shown that positive affect, negative affect, and life satisfaction are highly correlated^9^, and tend to load on a general SWB factor^10–12^ leading many authors to postulate a higher-order SWB factor. Consequently, it is common in the literature to encounter studies that use composite SWB scores as a way to examine global SWB.

Given the strongly-established link between SWB and positive outcomes, scholars have invested substantial effort in understanding why some people experience higher SWB than others. Toward this goal, research on the association between SWB and personality is highly relevant because it has the potential to uncover the basic biopsychosocial systems and processes that influence the nature of human subjective experience^13^. However, this depends on using models of personality for which there is strong evidence that individual personality traits correspond to unitary latent constructs with specified underlying psychological systems^14^^.^ Presently, lexical models of personality traits such as the ‘Big Five’^15,16^ and HEXACO^17^ models are dominant in research on personality and SWB, with studies often revealing weak to moderate associations (i.e., about 20% of variance) with extraversion and neuroticism^18^. However, to advance the state of the art it is important to consider alternative models like Cloninger’s psychobiological model of personality as measured by the Temperament and Character Inventory (TCI)^19,20^. This model is at least as good or better than other models in terms of predictive validity^21^ and the TCI has the benefit of measuring traits that are regulated by genetically, functionally, and developmentally distinct psychobiological systems of learning and memory^22–25^. However, despite the potential of this model for providing insights into SWB, it has not been as widely considered in research on this topic as the lexical models derived by the restrictive and questionable assumptions of linear factor analysis (although see^26–28^).”

Therefore, the overarching aim of this study was to examine the relationship between personality and SWB from the perspective of Cloninger’s psychobiological model. In particular, we aimed to add to current knowledge by using an approach that fully acknowledges the complex organizational hierarchy of human personality^25^. To this end, it is useful to briefly outline the features of Cloninger’s psychobiological model and recent evidence that validates its assumptions.

### The psychobiological model of personality

Cloninger’s psychobiological model of human personality is based on genomic and neuroimaging work that was not possible when the lexical tradition of personality assessment was developed using factor analytical techniques. It accounts well for the phenotypic variation measured by alternative models, and also provides a robust foundation in the genomics and neurobiology of learning and memory. According to Cloninger’s model of personality^19,20^, subjective experiences are dependent on organizations of psychobiological processes that underlie three distinct but interacting systems of learning and memory that evolved sequentially in human evolution: associative conditioning, intentionality, and self-awareness^24,29,30^. A concise summary of the genetics, neuroscience, and psychology of this research is available^31,32^.

### Temperament and character traits

Empirical findings robustly demonstrate a distinct domain of heritable and relatively stable aspects of personality that underlie and modulate the expression of basic emotions (i.e. temperament)^31,33^. Temperament involves individual differences in prelogical brain functions for associative conditioning of habits, attachments, and emotional reactivity. Individual differences in these brain functions are quantified by the TCI in terms of four empirically distinct dimensions: novelty seeking (impulsive, exploratory vs. deliberate, reserved), harm avoidance (fearful, pessimistic vs. risk taking, optimistic), reward dependence (friendly, sentimental vs. detached, objective), and persistence (determined, ambitious vs. easily discouraged, underachieving). Studies using functional neuroimaging have confirmed how individual differences in these four dissociable traits are associated with individual differences in the structure and function of brain regions involved in emotional functioning and associative conditioning^34^. Other studies provide strong neurophysiological, neuroanatomical, and biochemical evidence for the distinct psychobiological origins of these temperament traits^22,31,35–37^.

The psychobiological model of personality conceptualizes human personality also has a regulatory and cognitive domain in addition to the temperament domain. This self-regulatory domain of human character involves processes of intentionality and self-awareness that have been shown empirically to be genetically, psychologically, and developmentally distinct from temperament^31^. This domain of mental self-government includes executive functions that are intrapersonal (e.g., planning and foresight), legislative functions that are interpersonal (e.g., empathy and norms for cooperation), and judicial functions that are transpersonal (e.g., insight and intuitive evaluation of what is meaningful and good)^38^. Thus, the TCI quantifies individual differences in these processes in terms of three character dimensions: self-directedness (i.e., resourceful, purposeful, and responsible), cooperativeness (i.e. tolerant, helpful, and empathic) and self-transcendence (easily absorbed in flow states, meditative, and identifying with other people, nature, and what is sacred). Research indicates that character traits are as heritable as temperament traits^39^, and brain imaging studies have indicated that individual differences in character are reflected in differences in brain structure and function. Structurally, TCI character traits are correlated with local gray and white matter volumes in brain regions that are involved in self-reflection (self-directedness), empathizing (cooperation), and religious belief (self-transcendence)^40^.

Various past studies provide evidence that harm avoidance, persistence, and self-directedness may be particularly important for understanding SWB. For example, harm avoidance has shown to be significantly higher in participants with mood or anxiety disorders compared to those without, with the opposite pattern evident for self-directedness and persistence^41^. Prior work has also shown that adolescents reporting high positive affect and low negative affect have significantly lower harm avoidance and higher self-directedness than those reporting low positive affect and high negative affect^42^. More indirectly, a systematic review has indicated that well-being is most consistently associated with brain activation in the anterior cingulate cortex^43^, which serves to emotional and cognitive functions, such as regulating emotion in accord with goals and values, and is strongly correlated with individual differences in persistence^44^. However, it is worth noting that average associations estimated with linear methods can be weak or inconsistent because TCI traits are nonlinear in their functional effects.

### Multi-trait temperament and character profiles

Recent genomic studies using deep machine learning algorithms have uncovered that human subjective experience depends on complex interactions among the temperament traits, among the character traits, and between temperament and character, rather than on individual traits acting independently^22,23,25^. For example, there is extensive empirical support that genes code for different temperament configurations that describe the whole person^31,45^. Such temperament configurations include the ‘reliable’ profile (defined by high persistence and reward dependence, and low novelty seeking and harm avoidance), the ‘sensitive’ profile (high harm avoidance, reward dependence, and novelty seeking), and the ‘antisocial’ profile (high novelty seeking, low reward dependence and persistence)^22^. Similar latent profiles have been identified in independent samples^46,47^.

Beyond these major temperament types, recent studies using artificial classification methods (e.g., median split) have identified 16 configurations of high and low values for the four temperaments^48^. Studies have also identified and tested how multi-trait character profiles differ in well-being. Notably, a recent study by Zwir et al.^23^ identified distinct sets of genes that coded for five configurations of high and low values for character traits: three reflecting healthy personalities and two reflecting unhealthy personalities. Other studies have used artificial classification methods to cluster participants into the eight possible configurations of high and low values for the three character traits^26,28,49,50^. Research using latent profile analysis to identify naturally occurring characters has also identified these eight theoretical configurations^47^.

A growing number of studies have examined how multi-trait personality profiles relate to indicators of well-being, although most have focused on character because of its prominent self-regulatory role. This work has consistently show that physical, mental, and social well-being is strongest when all three character traits are high, and lowest when all three character traits are low^23,26,28,50,51^. These studies have also shown that the three TCI character traits make different non-linear contributions to positive affect, negative affect, and life satisfaction^26,27^. When only changing one character dimension in the configuration, higher life satisfaction was shown to be linked consistently to higher self-directedness; higher negative affect was linked to lower self-directedness (and sometimes lower cooperativeness); while higher positive affect was linked to higher levels of all three character traits (i.e. the creative character).

A smaller body of prior work has also shown that distinct temperament configurations also differ markedly in well-being. In particular, a temperament configuration of high reward dependence, high persistence, low novelty seeking, and low harm avoidance (the reliable temperament) has been linked to increased probability for well-being^22^. However, it is noteworthy that this work used a single composite index of well-being derived from each participant’s configuration of character dimensions to facilitate cross-cultural replication. While character profile is a valid indicator of overall physical, mental, and social well-being^26,52^, this approach does not distinguish between the three separable aspects of SWB. Therefore, there is a specific need to examine how people with distinct temperament profiles differ in positive affect, negative affect, and life satisfaction.

### Joint temperament-character networks

Research has shown that the full range of possible temperament profiles can occur with each character profile, although the probabilities of the different temperament-character profile combinations differ on average^25,53,54^. Recent behavioral-genetic research has identified three nearly disjoint phenotypic networks that account for the complex relations of temperament profiles with character profiles^25^. These three networks have been labelled as (a) the emotional-unreliable network, (b) the organized-reliable network, and (c) the creative-reliable network.

The first of these personality networks, the emotional-unreliable network, is strongly associated with a genotypic network for emotion regulation and social attachment (associative conditioning), and individuals classified in this network are typically emotionally reactive with weak capacity for rational self-government. Studies have shown this network is primarily comprised of people with apathetic or dependent character (low in self-directedness and cooperativeness) associated with sensitive or antisocial temperaments (high novelty seeking, low in persistence)^25,47,55^. The second personality network, the organized-reliable network, is strongly associated with genes for the regulation of intentional goal-setting (intentionality). People in this network are capable of resourceful productivity but are conventional, materialistic and practical, and not always compassionate or empathetic. Studies have found this network is mostly comprised of people with a reliable temperament associated with characters that are high in self-directedness and/or in cooperativeness^25,47,55^. Finally, the creative-reliable network is strongly associated with genes for episodic learning and autobiographic memory that allow for intuitive insight and creative imagination in the appraisal of values and theories (self-awareness). People in this third network primarily have a reliable temperament associated with a creative character^25,47,55^, are therefore capable of resourceful productivity and are more compassionate, helpful, intuitive, meditative, and creative. Because of reciprocal interactions among temperament and character–character regulates temperament while temperament biases perception and behavior–these adaptive networks are self-organizing^56^. Such self-organization implies that all individuals have the potential to develop a mature and coherent character that can regulate habits, attachments, and innate emotional tendencies to maintain a subjective state of calm awareness, resilience, and positive emotionality; that is, in other words, to experience SWB^19,57^.

These three phenotypic networks are each strongly correlated with a distinct genotypic network that regulates a different system of learning and memory, each of which is linked to distinct brain circuits. Specifically, the emotional-unreliable network is associated with clusters of 249 genes involved in emotion regulation and social attachment by associative conditioning, with the habitual responses to extracellular stimuli regulated by the ERP and PI3K molecular pathways. The organized-reliable network is associated with clusters of 438 genes for the regulation of intentional goal-setting (intentionality). Specifically, intentional self-control of the seeking of food and other goals involves the phospho-inositol/ Calcium second-messenger signaling system within cells. The creative-reliable network is associated with genes for episodic learning and autobiographic memory that allow for intuitive insight and creative imagination that extends a person’s perspective beyond their present place, time, and identity. Thus, creative self-awareness is associated with clusters of 574 genes, including 267 genes in modern human beings (mostly long-non-coding RNAs and microRNAs) that are not found in chimpanzee or Neanderthal genomes. The genes for self-awareness allow regulation of epigenetic modification of brain circuitry and co-expression of genes in particular brain regions that comprise networks for awareness and evaluation of life as a narrative that gives a person meaning and satisfaction. In this way, the personality features of each network can be considered prototypical of a major system of learning and memory. The structure of the personality traits, genetic clusters, and environmental influences are nearly separate from one another, but there is sufficient overlap to allow collaborative interactions to facilitate the integration of the three learning networks in a way that allows a person to bring their person’s habits in accord with their goals and values despite changing internal conditions and external situations. Detailed descriptions of the identification and replication of these networks and their evolution are presented in various articles^22–25,32^. The application of these findings in person-centered psychotherapy has been described using Plato’s Allegory of the Cave and his metaphors for rational self-government elsewhere^58–61^.

Prior work has shown that the three temperament-character networks–reflecting different integrated configurations of brain circuits for associative conditioning, intentionality and self-awareness–are highly correlated with indicators of well-being. For example, Zwir et al. found that the creative-reliable network was associated with the highest probability well-being, followed by the organized-reliable network and finally the emotional-unreliable network^25^. Prior works have also shown that the three networks differ in comic style (people in the creative-reliable network had a ‘lighter’ style)^47^, and virtues and character strengths (people in the creative-reliable network were more self-controlled, caring and inquisitive)^62^, among other indicators of adaptive functioning^55^*.*

### Study aims and hypotheses

Despite this current knowledge, researchers still know relatively little about how SWB is influenced (a) by non-linear and dynamic interactions among configurations of personality features in specific situations; (b) by the multidimensional configurations of temperament and of character domains; or (c) by the personality networks that maintain health and SWB despite environmental changes. Moreover, within the growing number of studies that are addressing these issues, most have focused on the multi-trait domain of character because of its prominent self-regulatory role^23,26–28,50^. These studies have consistently show that the physical, mental, and social well-being is strongest when all three character traits are high, and lowest when all three character traits are low. Beyond this focus on character, studies by Zwir et al. have shown that higher well-being was linked to the specific (“reliable”) configuration of high reward dependence and persistence, and low novelty seeking and harm avoidance^22^, and highest in the creative-reliable phenotypic network^25^. However, it is noteworthy that in order to facilitate cross-cultural replication these studies used a single composite index of well-being derived from each participant’s configuration of character dimensions. While character profiles are a valid indicator of overall physical, mental, and social well-being^26,52^, this approach does not distinguish between the individual components of SWB; namely, positive affect, negative affect, and life satisfaction. Therefore, work is needed to evaluate how configurations of temperament and character are related to the individual components of SWB.

By examining how positive affect, negative affect, and life satisfaction are associated with personality at these increasing levels of descriptive complexity, we aimed to provide richer insights into the psychobiological systems and processes underpinning the multidimensional phenomenon of SWB^22–25^.

Toward this goal, a first aim was to use a person-centered approach to explore how interactions among distinct combinations of (a) temperament traits, and (b) character traits are associated with positive affect, negative affect, and life satisfaction. By using non-linear statistical methods^26^ we aimed to add to a growing body of research by highlighting the need to recognize the complex nonlinearity of developmental processes. We did not make explicit predictions on which specific temperament or character profiles would be most strongly linked to the SWB components, or on how specific pairs of temperament or character profiles might differ, instead exploring the results to provide a broader understanding of the multidimensional nature of human adaptive functioning. However, based on various prior works we expected harm avoidance, persistence and self-directedness to present the strongest associations on average across SWB dimensions. We also anticipated that higher self-transcendence would be uniquely associated with higher positive affect (because people high in this trait are able to manifest joy from transpersonal identification with something greater than themselves^19^). Additionally, we broadly expected that the reliable temperament and the creative character would be associated with the highest levels of SWB dimensions overall.

A second major aim of this study was to test the associations of the individual components of SWB with three temperament-character networks that are distinct in their evolution, genetics, development, biopsychosocial functions, and phenotypes^24,25^, and that regulate the three major systems of learning and memory that have evolved in modern human beings in addition to general intelligence. Studies have shown that these networks differ markedly in composite measures of physical, mental, and social well-being. However, no study has considered whether these differences are consistent across the three separable aspects of SWB. Based on current evidence we were able to formulate two specific hypotheses. Firstly, we expected that the emotional-unreliable network would be associated with the lowest levels of positive affect and life satisfaction, and the highest negative affect. This is because research has often shown that the emotional reactivity of an unregulated temperament from low intentional self-control is linked to ill-health, low well-being, and maladaptive functioning^23,63^.

Secondly, we postulated that the creative-reliable network would generally be associated with higher SWB, but particularly with positive affect. We formulated this hypothesis because research shows the capacity for self-awareness through reflection and meditation activates molecular processes that promote an upward spiral of plasticity, virtue and effective functioning that are beneficial to the self and others, allowing for the manifestation of joy and positive emotions^23,64–66^. Indeed, studies have shown that capacity for self-awareness is linked to greater parasympathetic activity allowing individuals to operate in a state of calm awareness, and to function flexibly when confronted with challenges, thus maintaining state of positive emotionality^67^.

#### Hypothesis 1

The emotional-unreliable network will be associated with the lowest positive affect and life satisfaction, and highest negative affect.

#### Hypothesis 2

The creative-reliable network will be unique in its association with higher positive affect.

## Methods

### Participants

#### Adult sample

The first sample (Sample 1) comprised two moderately sized independent data sets that were pooled into one. Pooling two samples has the benefit of increasing statistical power and sample heterogeneity (due to design characteristics and sampling methods etc.)^68^. To obtain this convenience sample we approached undergraduate university students from several degree programs at the lead authors’ institution. Students agreeing to partake in the study were given survey packs to distribute to friends and family. Data were obtained for 767 individuals. Because of the sampling strategy, the sample age was skewed toward younger adults. The second data set comprised 400 adults who were recruited as part of a study on personality and religiousness. These adults were also recruited using a non-probabilistic chain referral sampling technique.

The initial pooled sample comprised 1167 adults. For data to be included in the final analysis we determined that participants must be ≥ 18 years (which excluded 12 individuals), to have responded to > 25% items from the study measures (which excluded 46 individuals), and to have responded correctly to all five attention-check items within the TCI-R (which excluded 212 individuals). Thus, the final sample comprised 897 adults (30% male, 70% female) aged between 18 and 88 years (*M* = 35.7, *SD* = 16.8).

### Adolescent sample

The second sample (Sample 2) was a sample of ninth graders participating in the third wave of a longitudinal study on student engagement in sustainable development. In Portugal, the 9th grade corresponds to the final year of basic education. Data collection for this third wave occurred during the COVID-19 pandemic between November 2020 and January 2021, which was period of widespread stress. In total, we obtained data for 1,823 individuals. However, for data to be included in the final analysis participants had to be < 18 years (excluding 8 individuals) and to have respond to > 25% items from the study measures (excluding 78 individuals). The JTCI does not include attention-check items. After exclusions, the final sample comprised 1,739 adults from 57 schools. Within this sample there were 756 boys (43%) and 849 girls (49%) with a mean age of 14.2 years (*SD* = 0.7).

## Measures

### Positive and negative affect

The emotional component of SWB was measured in Portuguese adults using the *Positive and Negative Affect Schedule* (PANAS)^69,70^. This instrument comprises 20 adjectives that describe emotional experiences: 10 positive (example item: “Excited”) and 10 negative (example item: “Afraid”). For each item, participants are asked to rate the extent to which they have felt the emotion over the last weeks on a scale from 1 (not at all or very little) to 5 (extremely). Omega coefficients were 0.89 and 0.90 for the positive affect and negative affect scales, respectively.

In adolescents, the emotional component of SWB was measured using the *Emotional Tonality Scale*, itself an adaptation of the PANAS. This instrument comprises 27 adjectives that describe emotional experiences: 12 positive (example item: “Excited”) and 15 negative (example item: “Afraid”). Like the PANAS, participants are asked to rate the extent to which they have felt the emotion over the last weeks on a scale from 1 (not at all or very little) to 5 (extremely). Omega coefficients were 0.88 and 0.91 for the positive affect and negative affect scales, respectively.

### Life satisfaction

The cognitive component of SWB was measured in adults using the *World Health Organization Quality-of-Life Scale* (WHOQOL-BREF). This 26-item scale was developed to assess “individuals’ perceptions of their position in life in the context of the culture and value systems in which they live and in relation to their goals, expectations, standards and concerns”^71^. In total, 24 of the 26 items are grouped within four domains: physical health, psychological health, social relationships, and physical environment. The remaining two items reflect global assessments of life satisfaction quality and health (example item: “How would you rate your quality of life”). All items are rated on a scale from 1 to 5, with higher scores reflecting increased well-being. We calculated a mean average score for this scale excluding item 26 because this item “How often do you have negative feelings such as blue mood, despair, anxiety, depression?” refers to the emotional, rather than cognitive, component of SWB. The omega coefficient for this scale was 0.91.

In adolescents, the cognitive component of SWB was measured in this sample using the six-item *Brief Multidimensional Students’ Life Satisfaction Scale*^72^. These items require respondents to judge their satisfaction with five specific life domains—family life, friends, school, oneself, and home–as well as life in general. Satisfaction is rated on a six-point scale from 0 (terrible) to 6 (fantastic). Research showed this scale has measurement invariance across 23 diverse populations^73^. The omega coefficient for this scale was 0.91.

### Personality

In adults, personality was measured using the European Portuguese *Temperament and Character Inventory* (TCI-R)^74,75^. The 240 items of this scale measure the behavioral and subjective aspects of how people respond in different situations. Four temperament dimensions quantify individual differences in associative conditioning and habitual behaviors: Novelty Seeking, Harm Avoidance, Reward Dependence, and Persistence. Three character dimensions capture the rational self-regulatory domain of personality: Self-Directedness, Cooperativeness, and Self-Transcendence. Omega coefficients for the seven scales ranged from 0.76 to 0.90.

Adolescents responded to the European Portuguese *Junior Temperament and Character Inventory* (JTCI)^76^, an adaptation of the TCI intended for youth aged 9 years and older^77^. This version of the JTCI has 127 items to capture the four temperament dimensions and three character dimensions of Cloninger’s psychobiological model of personality. All items are rated on a five-point scale from 1 (completely false) to 5 (completely true). Omega coefficients for the seven scales ranged from 0.68 to 0.89.

### Statistical analyses

We began by calculating correlations between all study variables. Because there was some indication that not all variables were normally distributed, we opted to calculate Spearman’s correlations coefficients. We considered correlation values ≥ 0.20 as being “practically” significant in terms of effect size^78^.

Next, to describe how dynamic intra-individual organizations of biopsychosocial processes relate to SWB we used a person-centered approach to analysis. We formed temperament profiles by dividing participants into groups reflecting those above and below the normative median^79^ for each of the four temperament traits, after excluding participants who were in the center of the distribution (45th to 55th percentile) for all four traits. This resulted in the 16 possible combinations of high and low values for novelty seeking, harm avoidance, reward dependence, and persistence shown in Table 1. The same procedure was repeated to group participants into the eight possible combinations of high and low character score on self-directedness, cooperativeness, and self-transcendence, again after excluding participants who were in the center of the distribution (45th to 55th percentile) for all three traits. To represent the three temperament-character networks we grouped participants into clusters of people expected to have low overall health (i.e., the emotional-unreliable cluster of people with character profiles including low self-directedness), intermediate health (i.e., the organized reliable cluster of people with characters that are high in self-directedness but not always high in cooperativeness and/or self-transcendence ), and high well-being (i.e., the creative reliable cluster of people with high self-directedness, cooperativeness and self-transcendence), as in prior works. The average age in years and gender distribution for each personality profile are in Supplementary Table 1.

**Table 1:** Frequency distributions of temperament profiles, character profiles, and temperament-character networks.

	Adult Sample		Adolescent Sample	
	*N*	%	*N*	%
Temperament profile				
nhrp “Independent”	19	2.1	68	3.9
nhrP	50	5.6	100	5.8
nhRp “Reliable”	15	1.7	17	1.0
nhRP	40	4.5	213	12.2
nHrp “Methodical”	85	9.5	109	6.3
nHrP	59	6.6	100	5.8
nHRp “Cautious”	28	3.1	67	3.9
nHRP	37	4.1	252	14.5
Nhrp “Adventurous”	40	4.5	81	4.7
NhrP	46	5.1	32	1.8
NhRp “Passionate”	25	2.8	15	0.9
NhRP	61	6.8	38	2.2
NHrp “Explosive”	78	8.7	178	10.2
NHrP	29	3.2	33	1.9
NHRp “Sensitive”	44	4.9	62	3.6
NHRP	43	4.8	50	2.9
*EXCLUDED*	198	22.1	324	18.6
Character Profile				
Sct “Apathetic”	116	12.9	341	19.6
scT “Disorganized”	123	13.7	139	8.0
sCt “Dependent”	39	4.3	78	4.5
sCT “Moody”	77	8.6	185	10.6
Sct “Bossy”	65	7.2	88	5.1
ScT “Fanatical”	33	3.7	61	3.5
SCt “Organized”	112	12.5	292	16.8
SCT “Creative”	117	13.0	407	23.4
*EXCLUDED*	215	24.0	148	8.5
Temperament-Character Network				
Emotional-Unreliable	355	39.6	743	42.7
Organized-Reliable	210	23.4	441	25.4
Creative-Reliable	117	13.0	407	23.4
*EXCLUDED*	215	24.0	148	8.5

N = high novelty seeking; n = low novelty seeking; H = high harm avoidance; h = low harm avoidance; R = high reward dependence; r = low reward dependence; P = high persistence; p = low persistence; S = high self-directedness; s = low self-directedness; C = high cooperativeness; c = low cooperativeness; T = high self-transcendence; t = low self-transcendence.

After forming personality profiles, we performed as series of robust ANOVAs^80,81^ based on 20% trimmed means to test differences in SWB dimensions across temperament profiles, character profiles, and temperament-character networks. Effect sizes for robust ANOVAs were assessed by calculating an explanatory measure of effect size (ξ)^82^ that does not require equal variance. Values of $\hat{\xi }$  = 0.10, 0.30, and 0.50 were set as thresholds for small, medium and large effect sizes^80^*.*Each robust ANOVA was followed by robust post-hoc tests, also based on 20% trimmed means. For these robust post-hoc comparisons, 95% confidence intervals for the possible effect sizes were corrected for the number of tests performed. All analyses were performed using R (version 4.1.2)^83^.

### Missing data

For adults, 91% of participants had no missing data for the PANAS and 93% had no missing data for the WHOQOL-BREF. Moreover, 95% of participants had < 1% missing data for the TCI-R. For adolescents, 99% of participants had no missing data for the PANAS and > 99% of participants had no missing data for the BMSLSS. 98% of adolescents had no missing data for the JTCI. We imputed all missing data using a predictive mean matching single imputation method.

### Ethical declarations

To ensure adherence to the Declaration of Helsinki, the study protocols for each sample were approved by the ethics committee of the Centro de Investigação em Psicologia para o Desenvolvimento (Ref: CIPD/2122/PERS/3) or the ethics committee at Universidade Lusíada Porto (Ref: UL/CE/CIPD/2207). All research methods were performed in accordance with relevant guidelines/regulations. Informed consent was obtained from all participants prior to the study. For participants under the age of 18 years, informed consent was also obtained from a legal guardian.

## Results

### Descriptive statistics

A detailed summary of descriptive statistics for both samples is presented in Supplementary Materials. However, while none of the variables presented strong skew or kurtosis (all values <|1|), it was apparent that scores for negative affect were skewed toward the lower end of the scale (skew = 0.91).

### Correlational analyses

Table 2 presents the correlations among all study variables in both samples. Because the samples were large, it was unsurprising that most TCI traits correlated significantly with SWB dimensions. It was also notable that the findings were generally consistent across the two samples.

**Table 2:** *Correlations between subjective well-being dimensions and TCI temperament and character traits for adults (below the diagonal; N* = *897) and adolescents (above the diagonal; N* = *1739).*

	1	2	3	4	5	6	7	8	9	10
Positive affect		0.06*	0.40*	0.01	− 0.20*	0.24*	0.30*	0.33*	0.18*	0.24*
Negative affect	− 0.06		− 0.44*	0.32*	0.42*	− 0.16*	− 0.24*	− 0.46*	− 0.24*	0.12*
Life satisfaction	0.47*	− 0.39*		− 0.20*	− 0.33*	0.34*	0.33*	0.53*	0.31*	0.11*
Novelty seeking	0.12*	0.05	0.05		0.03	− 0.25*	− 0.47*	− 0.46*	− 0.49*	0.04
Harm avoidance	− 0.39*	0.40*	− 0.44*	− 0.23*		0.00	− 0.14*	− 0.40*	0.00	0.21*
Reward dependence	0.22*	− 0.07*	0.21*	0.19*	− 0.12*		0.44*	0.43*	0.60*	0.22*
Persistence	0.41*	− 0.05	0.31*	− 0.11*	− 0.31*	0.15*		0.64*	0.61*	0.18*
Self-directedness	0.19*	− 0.48*	0.46*	− 0.15*	− 0.48*	0.17*	0.29*		0.58*	0.12*
Cooperativeness	0.16*	− 0.18*	0.24*	− 0.12*	− 0.14*	0.48*	0.20*	0.45*		0.34*
Self-transcendence	0.26*	0.11*	− 0.02	0.09*	− 0.07*	0.24*	0.29*	− 0.12*	0.14*	

Values are Spearman’s correlation coefficients. *p < .05.

#### Positive affect

Consistent with our expectations the correlation coefficients indicated harm avoidance had an inverse negative relationship with positive affect in both samples (R = −0.20 and −0.39) thus contributing to 4% and 15% of the variance in adolescents and adults, respectively. Next, we found that persistence had a positive relationship with positive affect (R = 0.30 and 0.41), contributing to more variation than harm avoidance (9% and 17% respectively). Finally, we observed that self-directedness was the TCI trait most strongly correlated with positive affect in adolescents (R = 0.33), contributing to 11% of the variance. However, in adults self-directedness has a less strong linear relationship with positive affect (R = 0.19) than self-transcendence (R = 0.26) and reward dependence (R = 0.22).

#### Negative affect

It was evident from Table 2 that in both samples harm avoidance (*R* = 0.42 and 0.40) and self-directedness (*R* = −0.46 and −0.48) had the strongest linear associations with negative affect. Thus, self-directedness appeared to make a moderate contribution to negative affect, accounting for 21% and 23% of the variance, respectively. An interesting finding was that for adolescents novelty seeking had a positive linear association with negative affect (*R* = 0.32) while in adults this relationship was close to zero (*R* = 0.05). It was also noteworthy that persistence was negatively correlated with negative affect in adolescents (*R* = −0.24) but close to uncorrelated in adults (*R* = −0.05).

#### Life satisfaction

Mirroring the results for negative affect, it was evident in Table 2 that life satisfaction was most strongly associated with self-directedness (*R* = 0.53 and 0.46), accounting for 28% and 21% of the variance, respectively. In both samples life satisfaction was also negatively associated with harm avoidance (*R* = −0.33 and -0.44), and positively associated with persistence (*R* = 0.33 and 0.31). Unlike for negative affect, life satisfaction was shown to have a positive association with cooperativeness (*R* = 0.31 and 0.24) and reward dependence (*R* = 0.34 and 0.21).

## Temperament results

### Differences among temperament profiles

**Figure 1:**
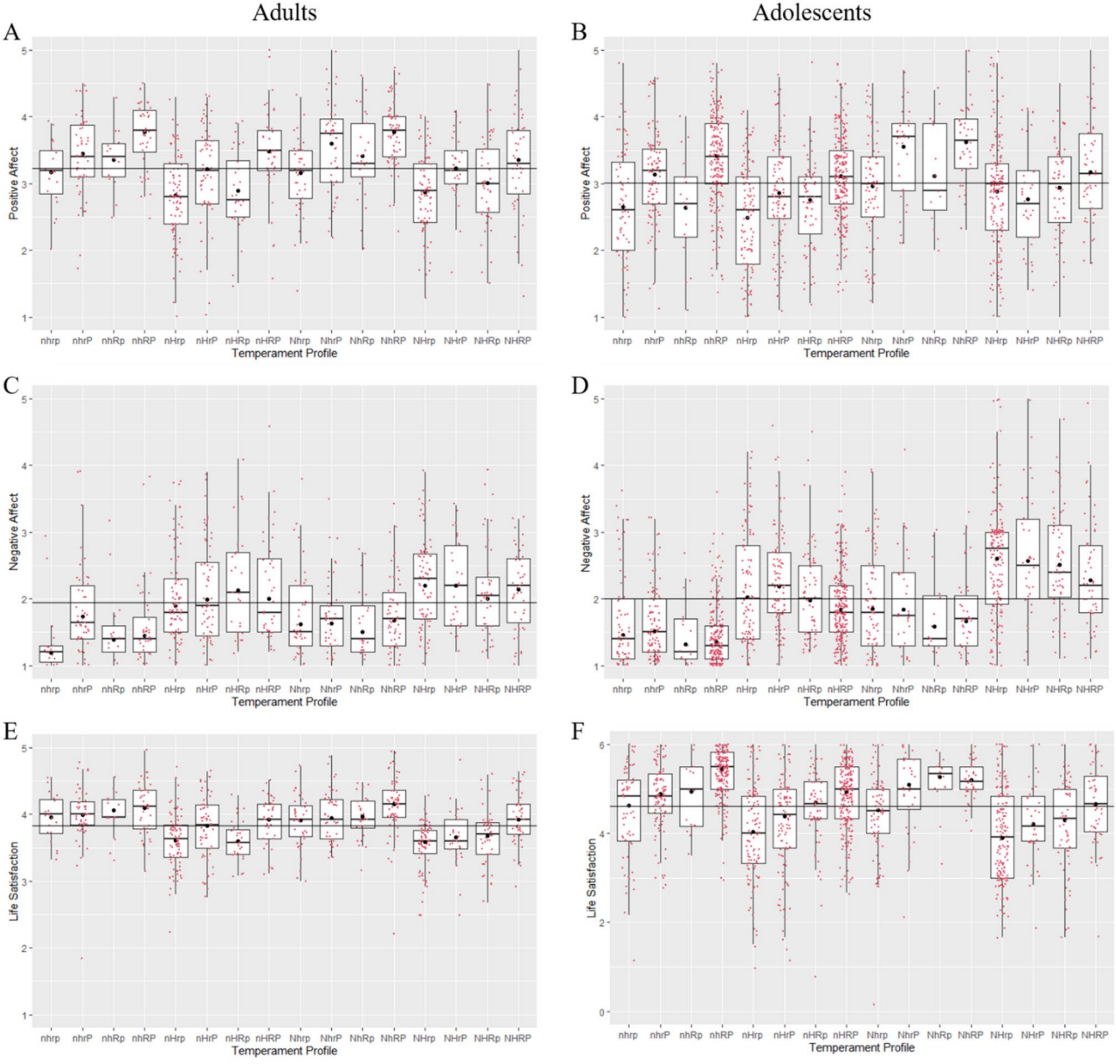
*Boxplots with superimposed 20% trimmed means (black dots) for positive affect, negative affect, and life satisfaction across temperament profiles. Note.* (**A, B**) Differences for Positive Affect. (**C, D**) Differences for Negative Affect. (**E, F**) Differences for Life satisfaction. Horizontal lines represent the grand mean for each variable.

Mean scores for positive affect, negative affect, and life satisfaction (see Fig. 1) were compared among people in the 16 temperament profiles using robust ANOVAs. These indicated the between-subjects effect of temperament profile was significant for positive affect (adults: *p* < 0.001, $\hat{\xi }$ = 0.52; adolescents: *p* < 0.001, $\hat{\xi }$ = 0.46); negative affect (adults: *p* < 0.001, $\hat{\xi }$ = 0.49; adolescents: *p* < 0.001, $\hat{\xi }$ = 0.52); and life satisfaction (adults: *p* < 0.001, $\hat{\xi }$ = 0.51; adolescents: *p* < 0.001, $\hat{\xi }$ = 0.51).

### Non-linear analysis of temperament dimensions

We evaluated the non-linear associations between temperament traits and SWB dimensions by examining selected paired comparisons from robust post-hoc tests, which are summarized in Table 3 (Adults sample) and Table 4 (Adolescent sample). These profiles capture the 16 possible configurations of novelty seeking (N = high novelty seeking; n = low novelty seeking), harm avoidance (H = high harm avoidance; h = low harm avoidance), reward dependence (R = high reward dependence; r = low reward dependence), and persistence (P = high persistence; p = low persistence). Mean differences between pairs of temperament profiles (e.g. nhrp vs. nhrP) were interpreted as statistically significant when their associated confidence intervals (CIs) did not include the value zero.

**Table 3:** Robust post-hoc comparisons between temperament profiles testing non-linear effects of temperament traits in adults.

Paired comparison	Positive affect		Negative affect		Life satisfaction	
	Difference	[95% CI]	Difference	[95% CI]	Difference	95% CI
Novelty seeking						
nhrp vs. Nhrp	− 0.01	[− 0.57, 0.54]	0.42	[− 0.01, 0.86]	− 0.05	[− 0.47, 0.36]
nhrP vs. NhrP	0.15	[− 0.30, 0.60]	− 0.11	[− 0.53, 0.31]	− 0.05	[− 0.34, 0.24]
nhRp vs. NhRp	0.06	[− 0.58, 0.70]	0.11	[− 0.45, 0.68]	− 0.09	[− 0.44, 0.25]
nhRP vs. NhRP	0.00	[− 0.40, 0.39]	0.23	[− 0.13, 0.59]	0.06	[− 0.24, 0.37]
nHrp vs. NHrp	0.06	[− 0.30, 0.42]	0.30	[− 0.08, 0.68]	− 0.03	[− 0.24, 0.19]
nHrP vs. NHrP	0.01	[− 0.44, 0.45]	0.20	[− 0.55, 0.95]	− 0.18	[− 0.51, 0.16]
nHRp vs. NHRp	0.12	[− 0.50, 0.74]	− 0.12	[− 0.88, 0.64]	0.08	[− 0.21, 0.37]
nHRP vs. NHRP	− 0.12	[− 0.67, 0.42]	0.13	[− 0.55, 0.82]	0.00	[− 0.33, 0.34]
Harm avoidance						
nhrp vs. nHrp	− 0.36	[− 0.89, 0.18]	**0.71**	**[0.39, 1.03]**	− 0.35	[− 0.74, 0.05]
nhrP vs. nHrP	− 0.23	[− 0.65, 0.19]	0.25	[− 0.27, 0.78]	− 0.17	[− 0.45, 0.12]
nhRp vs. nHRp	− 0.47	[− 1.09, 0.16]	0.74	[− 0.02, 1.50]	** − 0.46**	**[− 0.77, − 0.15]**
nhRP vs. nHRP	− 0.30	[− 0.77, 0.17]	0.56	[− 0.06, 1.19]	− 0.17	[− 0.52, 0.18]
Nhrp vs. NHrp	− 0.29	[− 0.69, 0.12]	**0.59**	**[0.11, 1.06]**	** − 0.32**	**[− 0.60, − 0.04]**
NhrP vs. NHrP	− 0.37	[− 0.85, 0.10]	0.56	[− 0.13, 1.25]	− 0.29	[− 0.63, 0.05]
NhRp vs. NHRp	− 0.41	[− 1.04, 0.23]	0.51	[− 0.04, 1.06]	− 0.29	[− 0.62, 0.04]
NhRP vs. NHRP	− 0.42	[− 0.91, 0.07]	0.47	[− 0.02, 0.95]	− 0.23	[− 0.51, 0.05]
Reward dependence						
nhrp vs. nhRp	0.18	[− 0.43, 0.79]	0.20	[− 0.31, 0.70]	0.10	[− 0.32, 0.52]
nhrP vs. nhRP	0.33	[− 0.11, 0.76]	− 0.29	[− 0.69, 0.10]	0.10	[− 0.21, 0.41]
nHrp vs. nHRp	0.07	[− 0.49, 0.63]	0.22	[− 0.49, 0.93]	− 0.01	[− 0.27, 0.25]
nHrP vs. nHRP	0.26	[− 0.20, 0.72]	0.01	[− 0.69, 0.71]	0.10	[− 0.24, 0.44]
Nhrp vs. NhRp	0.26	[− 0.34, 0.85]	− 0.12	[− 0.64, 0.41]	0.06	[− 0.29, 0.41]
NhrP vs. NhRP	0.17	[− 0.25, 0.59]	0.04	[− 0.34, 0.43]	0.22	[− 0.07, 0.50]
NHrp vs. NHRp	0.13	[− 0.34, 0.61]	− 0.20	[− 0.69, 0.30]	0.09	[− 0.16, 0.35]
NHrP vs. NHRP	0.13	[− 0.41, 0.66]	− 0.05	[− 0.79, 0.68]	0.28	[− 0.05, 0.61]
Persistence						
nhrp vs. nhrP	0.28	[− 0.26, 0.82]	**0.55**	**[0.18, 0.91]**	0.04	[− 0.36, 0.43]
nhRp vs. nhRP	0.42	[− 0.11, 0.96]	0.06	[− 0.45, 0.57]	0.04	[− 0.31, 0.38]
nHrp vs. nHrP	0.40	[− 0.01, 0.82]	0.09	[− 0.41, 0.60]	0.21	[− 0.07, 0.50]
nHRp vs. nHRP	**0.59**	**[0.01, 1.18]**	− 0.12	[− 0.95, 0.71]	**0.32**	**[0.01, 0.64]**
Nhrp vs. NhrP	0.44	[− 0.03, 0.92]	0.02	[− 0.46, 0.49]	0.04	[− 0.30, 0.37]
NhRp vs. NhRP	0.36	[− 0.21, 0.92]	0.18	[-0.28, 0.64]	0.19	[− 0.11, 0.50]
NHrp vs. NHrP	0.35	[-0.05, 0.76]	− 0.01	[− 0.70, 0.68]	0.06	[− 0.22, 0.35]
NHRp vs. NHRP	0.35	[-0.24, 0.93]	0.13	[-0.43, 0.70]	0.25	[− 0.06, 0.56]

Differences are differences in trimmed means ($\hat{\psi }$). Cells highlighted in bold are significant at *p* < .05.

**Table 4:** Robust post-hoc comparisons between temperament profiles testing non-linear effects of temperament traits in adolescents.

	Positive affect		Negative affect		Life satisfaction	
Paired Comparison	Difference	95% CI	Difference	95% CI	Difference	95% CI
Novelty seeking						
nhrp vs. Nhrp	0.30	[− 0.20, 0.81]	0.40	[− 0.09, 0.89]	− 0.10	[− 0.72, 0.51]
nhrP vs. NhrP	0.42	[− 0.12, 0.96]	0.32	[− 0.34, 0.99]	0.21	[− 0.49, 0.91]
nhRp vs. NhRp	0.47	[− 0.89, 1.82]	0.27	[− 0.52, 1.07]	0.34	[− 0.92, 1.59]
nhRP vs. NhRP	0.22	[− 0.22, 0.65]	0.31	[− 0.07, 0.69]	− 0.23	[− 0.52, 0.06]
nHrp vs. NHrp	**0.40**	**[0.02, 0.78]**	**0.58**	**[0.18, 0.98]**	− 0.14	[− 0.67, 0.39]
nHrP vs. NHrP	− 0.09	[− 0.84, 0.66]	0.38	[− 0.31, 1.08]	− 0.17	[− 0.92, 0.57]
nHRp vs. NHRp	0.19	[− 0.27, 0.64]	**0.54**	**[0.05, 1.03]**	− 0.39	[− 1.00, 0.22]
nHRP vs. NHRP	0.06	[− 0.40, 0.53]	**0.44**	**[0.00, 0.89]**	− 0.26	[− 0.80, 0.28]
Harm avoidance						
nhrp vs. nHrp	− 0.17	[− 0.68, 0.34]	**0.58**	**[0.13, 1.02]**	− 0.59	[− 1.26, 0.07]
nhrP vs. nHrP	− 0.27	[− 0.63, 0.08]	**0.66**	**[0.33, 1.00]**	** − 0.50**	**[− 0.96, − 0.05]**
nhRp vs. nHRp	0.11	[− 0.79, 1.01]	**0.66**	**[0.10, 1.22]**	− 0.24	[− 1.49, 1.00]
nhRP vs. nHRP	** − 0.30**	**[− 0.52, − 0.09]**	**0.49**	**[0.33, 0.65]**	** − 0.51**	**[− 0.71, − 0.30]**
Nhrp vs. NHrp	− 0.07	[− 0.45, 0.30]	**0.75**	**[0.31, 1.20]**	** − 0.63**	**[− 1.10, − 0.16]**
NhrP vs. NHrP	− 0.78	[− 1.60, 0.04]	0.72	[− 0.13, 1.58]	** − 0.89**	**[− 1.76, − 0.01]**
NhRp vs. NHRp	− 0.17	[− 1.47, 1.13]	**0.93**	**[0.16, 1.69]**	** − 0.97**	**[− 1.63, − 0.31]**
NhRP vs. NHRP	− 0.46	[− 1.03, 0.12]	**0.62**	**[0.09, 1.15]**	− 0.54	[− 1.11, 0.02]
Reward dependence						
nhrp vs. nhRp	− 0.01	[− 0.92, 0.90]	− 0.14	[− 0.68, 0.41]	0.30	[− 0.95, 1.56]
nhrP vs. nhRP	**0.28**	**[0.00, 0.56]**	− 0.17	[− 0.41, 0.07]	**0.55**	**[0.27, 0.84]**
nHrp vs. nHRp	0.27	[− 0.16, 0.70]	− 0.05	[− 0.53, 0.42]	**0.65**	**[0.14, 1.17]**
nHrP vs. nHRP	0.25	[− 0.06, 0.57]	** − 0.34**	**[− 0.63, − 0.06]**	**0.55**	**[0.13, 0.97]**
Nhrp vs. NhRp	0.15	[− 1.15, 1.46]	− 0.27	[− 1.04, 0.51]	**0.75**	**[0.21, 1.28]**
NhrP vs. NhRP	0.07	[− 0.54, 0.68]	− 0.18	[− 0.88, 0.52]	0.12	[− 0.58, 0.82]
NHrp vs. NHRp	0.06	[− 0.35, 0.47]	− 0.09	[− 0.52, 0.34]	0.40	[− 0.22, 1.03]
NHrP vs. NHRP	0.40	[− 0.40, 1.20]	− 0.28	[− 1.03, 0.47]	0.46	[− 0.34, 1.26]
Persistence						
nhrp vs. nhrP	**0.48**	**[0.02, 0.94]**	0.07	[− 0.30, 0.43]	0.25	[− 0.34, 0.83]
nhRp vs. nhRP	0.77	[− 0.14, 1.67]	0.03	[− 0.49, 0.56]	0.50	[− 0.75, 1.75]
nHrp vs. nHrP	0.37	[− 0.05, 0.79]	0.16	[− 0.27, 0.58]	0.34	[− 0.22, 0.90]
nHRp vs. nHRP	**0.36**	**[0.02, 0.69]**	− 0.14	[− 0.49, 0.22]	0.23	[− 0.12, 0.59]
Nhrp vs. NhrP	**0.60**	**[0.02, 1.17]**	− 0.01	[− 0.73, 0.71]	0.56	[− 0.16, 1.28]
NhRp vs. NhRP	0.52	[− 0.78, 1.82]	0.07	[− 0.69, 0.84]	− 0.07	[− 0.58, 0.44]
NHrp vs. NHrP	− 0.11	[− 0.85, 0.62]	− 0.04	[− 0.73, 0.64]	0.30	[− 0.43, 1.03]
NHRp vs. NHRP	0.23	[− 0.31, 0.78]	− 0.23	[− 0.78, 0.32]	0.36	[− 0.36, 1.08]

Differences are differences in trimmed means ($\hat{\psi }$). Cells highlighted in bold are significant at *p* < .05.

#### Positive affect

Overall, the two tables indicated that most paired comparisons were non-significant. However, an important observation was a trend for higher positive affect with higher persistence; with three significant contrasts in the adolescent sample and one significant contrast in the adult sample. Notably, in both samples, persistence was significantly associated with higher positive affect when comparing the higher vs. lower persistence variants of the nHR “Cautious” temperament.

#### Negative affect

The non-linear interactions of temperament traits for negative affect were not simply the opposite of those observed for positive affect. The most relevant finding was that higher harm avoidance was associated with higher negative affect for seven of the eight possible configurations of novelty seeking, reward dependence and persistence in adolescents. Fewer contrasts were significant for the adult sample, although the trend was in the same direct as the adolescents. A second noteworthy finding was that most comparisons between profiles differing in persistence were non-significant. Thirdly, for adolescents it was evident that higher novelty seeking was associated with higher negative affect in three contrasts where harm avoidance was high (nHrp vs. NHrp; nHRp vs. NHRp; and nHRP vs. NHRP).

#### Life satisfaction

Finally, there was a trend in both adults and adolescents for lower life satisfaction in profiles with higher harm avoidance. For adolescents, five of the eight comparisons were significant, with mean differences ranging from −0.50 to −0.97, while for adults two comparisons were significant (mean differences of −0.32 and −0.46). For adolescents only, there was an indication that higher reward dependence was associated with higher life satisfaction. In particular, this association was significant in three of the four comparisons where novelty seeking was low (although also in one comparison where novelty seeking was high).

## Character results

### Differences among character profiles

**Figure 2:**
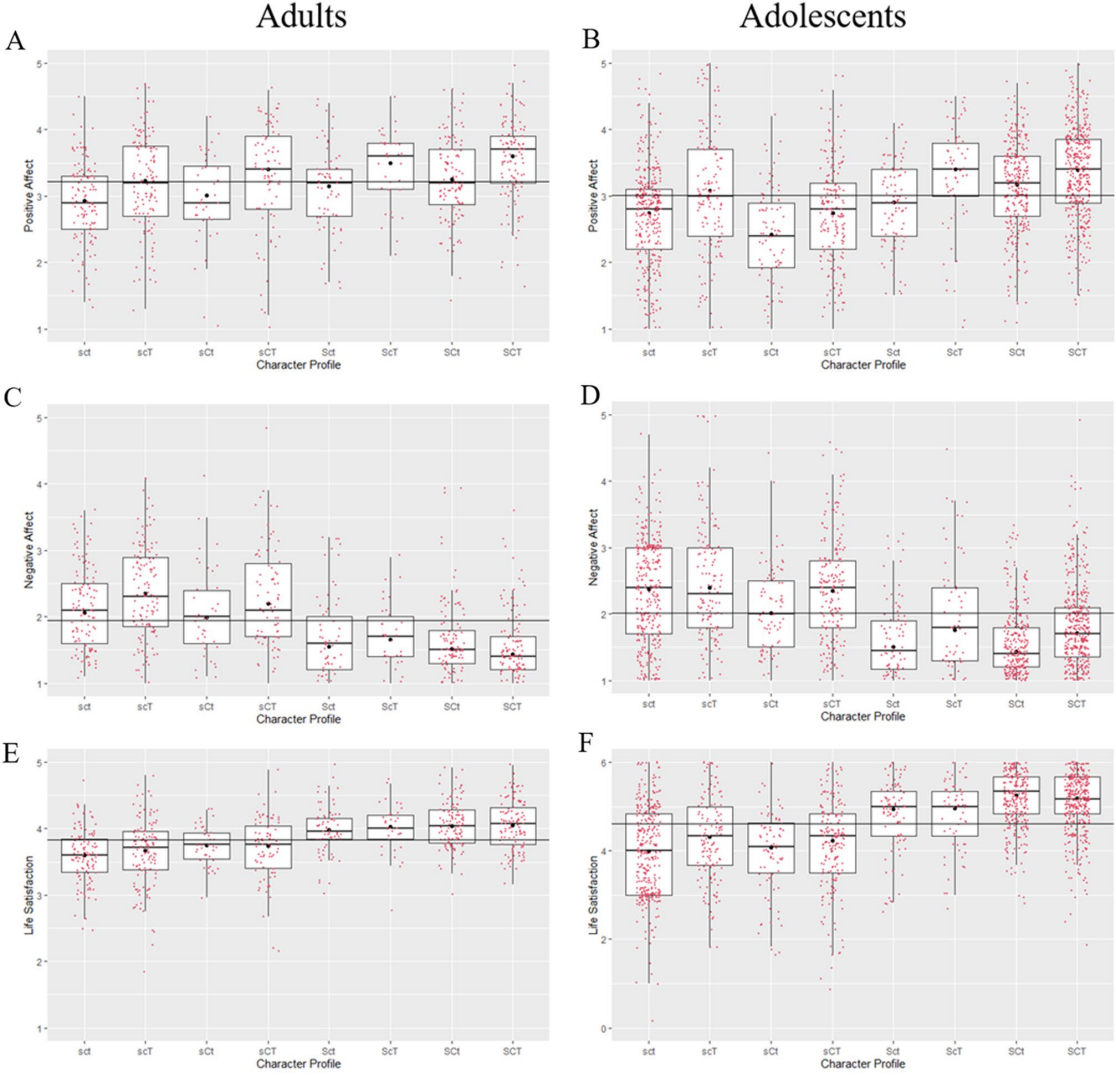
*Boxplots with superimposed 20% trimmed means (black dots) for positive affect, negative affect, and life satisfaction across character profiles. Note.* (**A, B**) Differences for Positive Affect. (**C, D**) Differences for Negative Affect. (**E, F**) Differences for Life satisfaction. Horizontal lines represent the grand mean for each variable.

Figure 2. presents participants’ scores for positive affect, negative affect, and life satisfaction grouped by the 8 distinct character profiles formed of self-directedness (S = high self-directedness; s = low self-directedness), cooperativeness (C = high cooperativeness; c = low cooperativeness), and self-transcendence (T = high self-transcendence; t = low self-transcendence). Robust ANOVAs indicated the between-subjects effect of character profile was significant for positive affect (adults: *p* < 0.001, $\hat{\xi }$ = 0.37; adolescents: *p* < 0.001, $\hat{\xi }$ = 0.43); negative affect (adults: *p* < 0.001, $\hat{\xi }$ = 0.50; adolescents: *p* < 0.001, $\hat{\xi }$ = 0.46); and life satisfaction (adults: *p* < 0.001, $\hat{\xi }$ = 0.50; adolescents: *p* < 0.001, $\hat{\xi }$ = 0.50).

### Non-linear analysis of character dimensions

Paired comparisons to test the associations of each character dimension with SWB dimensions while controlling for the others (e.g. sct vs. scT) are summarized in Table 5 (Adult sample) and Table 6 (Adolescent sample).

**Table 5:** Robust post-hoc comparisons between character profiles testing non-linear effects of each character trait on positive affect, negative affect, and life satisfaction in adults.

	Positive affect		Negative affect		Life satisfaction	
	Difference	[95% CI]	Difference	[95% CI]	Difference	[95% CI]
Self-directedness						
sct vs. Sct	0.22	[− 0.10, 0.54 ]	** − 0.51**	**[− 0.87, − 0.15 ]**	**0.38**	**[ 0.20, 0.56 ]**
scT vs. ScT	**0.57**	**[ 0.13, 1.02 ]**	** − 0.41**	**[− 0.78, − 0.04 ]**	**0.43**	**[ 0.19, 0.67 ]**
sCt vs. SCt	**0.33**	**[ 0.05, 0.62 ]**	** − 0.54**	**[− 0.81, − 0.27 ]**	**0.44**	**[ 0.28, 0.61 ]**
sCT vs. SCT	**0.67**	**[ 0.41, 0.93 ]**	** − 0.62**	**[− 0.88, − 0.36 ]**	**0.45**	**[ 0.28, 0.63 ]**
Cooperativeness						
sct vs. sCt	0.09	[− 0.32, 0.50 ]	− 0.07	[− 0.52, 0.38 ]	0.16	[− 0.06, 0.37 ]
scT vs. sCT	0.17	[− 0.22, 0.57 ]	− 0.15	[− 0.52, 0.22 ]	0.07	[− 0.17, 0.30 ]
Sct vs. SCt	0.12	[− 0.21, 0.45 ]	− 0.03	[− 0.35, 0.28 ]	0.06	[− 0.11, 0.24 ]
ScT vs. SCT	0.10	[− 0.34, 0.53 ]	− 0.21	[− 0.55, 0.12 ]	0.02	[− 0.23, 0.27 ]
Self-transcendence						
sct vs. scT	**0.30**	**[ 0.00, 0.60 ]**	0.29	[− 0.03, 0.61 ]	0.08	[− 0.11, 0.27 ]
sCt vs. sCT	0.39	[− 0.09, 0.86 ]	0.21	[− 0.27, 0.69 ]	− 0.01	[− 0.27, 0.24 ]
Sct vs. ScT	0.36	[− 0.11, 0.83 ]	0.10	[− 0.31, 0.50 ]	0.05	[− 0.20, 0.30 ]
SCt vs. SCT	**0.34**	**[ 0.07, 0.61 ]**	− 0.08	[− 0.28, 0.11 ]	0.01	[− 0.16, 0.18 ]

Differences are differences in trimmed means ($\hat{\psi }$). Highlighted in bold are significant at *p* < .05.

**Table 6:** Robust post-hoc comparisons between character profiles testing non-linear effects of each character trait on positive affect, negative affect, and life satisfaction in adolescents.

	Positive affect		Negative affect		Life satisfaction	
	Difference	[95% CI]	Difference	[95% CI]	Difference	[95% CI]
Self-directedness						
sct vs. Sct	0.16	[− 0.12, 0.44 ]	** − 0.86**	**[− 1.12, − 0.60 ]**	**0.96**	**[ 0.60, 1.32 ]**
scT vs. ScT	**0.65**	**[ 0.31, 1.00 ]**	** − 0.60**	**[− 1.01, − 0.19 ]**	**0.98**	**[ 0.55, 1.40 ]**
sCt vs. SCt	**0.43**	**[ 0.24, 0.61 ]**	** − 0.93**	**[− 1.12, − 0.73 ]**	**1.27**	**[ 1.01, 1.54 ]**
sCT vs. SCT	**0.64**	**[ 0.47, 0.81 ]**	** − 0.65**	**[− 0.85, − 0.46 ]**	**1.20**	**[ 0.95, 1.45 ]**
Cooperativeness						
sct vs. sCt	** − 0.33**	**[− 0.65, − 0.02 ]**	** − 0.36**	**[− 0.70, − 0.01 ]**	0.09	[− 0.33, 0.50 ]
scT vs. sCT	** − 0.33**	**[− 0.67, 0.00 ]**	− 0.04	[− 0.38, 0.30 ]	− 0.08	[− 0.48, 0.33 ]
Sct vs. SCt	0.27	[− 0.01, 0.54 ]	− 0.07	[− 0.29, 0.16 ]	**0.31**	**[ 0.00, 0.62 ]**
ScT vs. SCT	− 0.02	[− 0.35, 0.32 ]	− 0.05	[− 0.44, 0.34 ]	0.22	[− 0.16, 0.61 ]
Self-transcendence						
sct vs. scT	**0.33**	**[ 0.02, 0.64 ]**	0.03	[− 0.29, 0.35 ]	0.33	[− 0.07, 0.73 ]
sCt vs. sCT	0.33	[− 0.01, 0.67 ]	0.34	[− 0.02, 0.70 ]	0.17	[− 0.25, 0.59 ]
Sct vs. ScT	**0.49**	**[ 0.10, 0.89 ]**	0.26	[− 0.16, 0.68 ]	0.01	[− 0.44, 0.47 ]
SCt vs. SCT	**0.21**	**[ 0.04, 0.38 ]**	**0.27**	**[ 0.14, 0.41 ]**	− 0.07	[− 0.24, 0.10 ]

Differences are differences in trimmed means ($\hat{\psi }$). Highlighted in bold are significant at *p* < .05.

#### Positive affect

A first major finding from the two tables was that higher positive affect was associated with higher self-directedness for three of the four possible configurations of cooperativeness and self-transcendence. For both samples, higher self-directedness was not significantly associated with higher positive affect when cooperativeness and self-transcendence were both low (sct vs. Sct). A second noteworthy result was that higher positive affect was associated with higher self-transcendence, with both samples showing significant differences when self-directedness and cooperativeness were both low (sct vs. scT), and both high (SCt vs. SCT).

#### Negative affect

Most notably, we observed that higher self-directedness was consistently associated with lower negative affect, with all contrasts significantly different in both samples. Cooperativeness and self-transcendence, in contrast, did not appear to show strong associations with negative affect. That said, an interesting finding was that for adolescents higher self-transcendence was linked to higher negative affect for the SCt vs. SCT contrast.

#### Life satisfaction

Like negative affect, the most notable finding for life satisfaction was the strong consistent association with self-directedness in both samples. The results in Tables 5 and 6 also indicated that life satisfaction was largely not associated with cooperativeness or self-transcendence.

### Differences among personality networks

**Figure 3:**
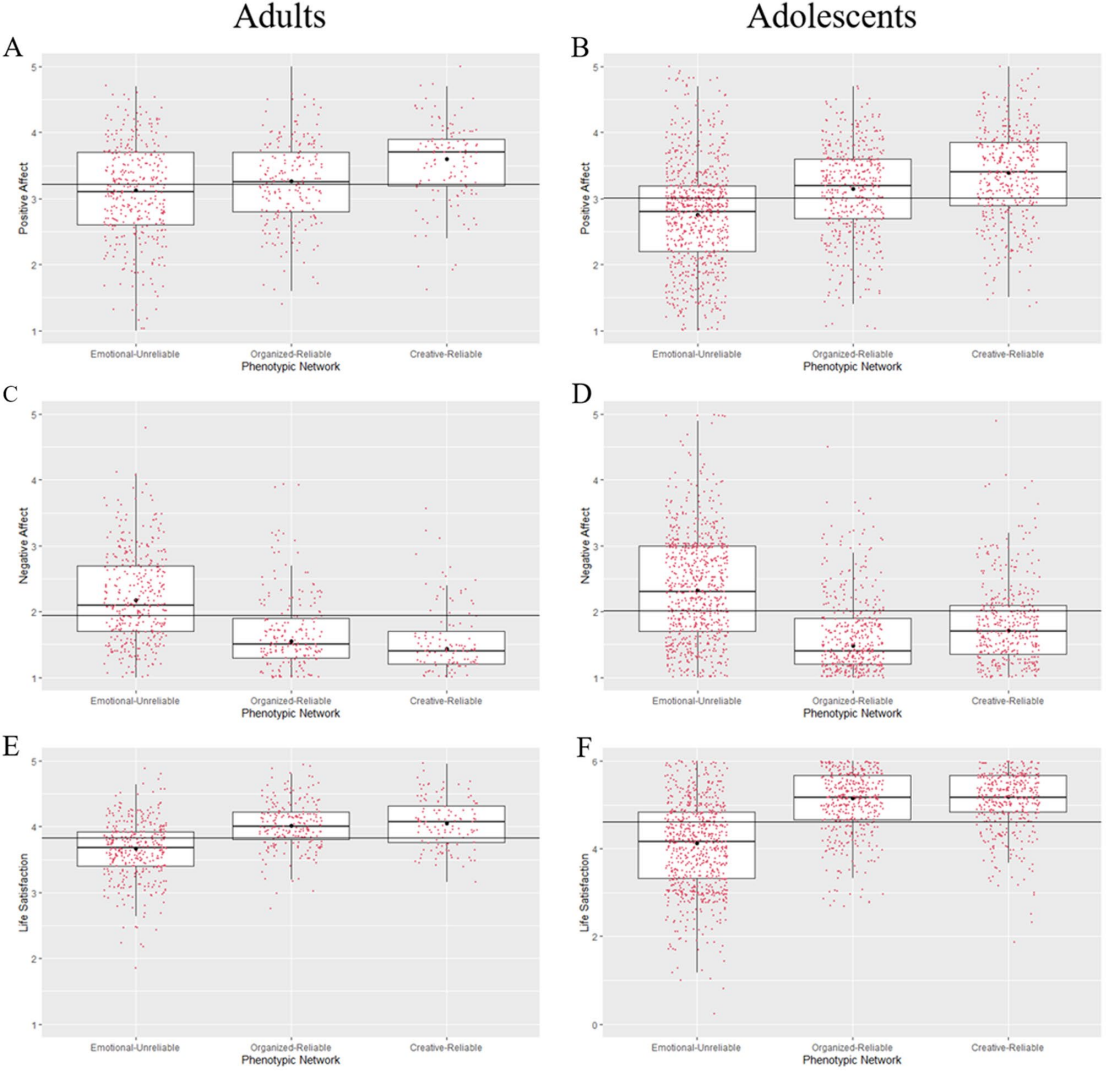
*Boxplots with superimposed 20% trimmed means (black dots) for positive affect, negative affect, and life satisfaction across temperament-character networks. Note.* (**A, B**) Differences for Positive Affect. (**C, D**) Differences for Negative Affect. (**E, F**) Differences for Life satisfaction. Horizontal lines represent the grand mean for each variable.

Figure 3 presents participants’ scores for positive affect, negative affect, and life satisfaction grouped by the three personality networks. There was a large convergence between the results for adults and adolescents. Robust ANOVAs showed significant differences among the networks for positive affect (adults: *p* < 0.001, $\hat{\xi }$ = 0.34; adolescents: *p* < 0.001, $\hat{\xi }$ = 0.41); negative affect (adults: *p* < 0.001, $\hat{\xi }$ = 0.62; adolescents: *p* < 0.001, $\hat{\xi }$ = 0.54); and life satisfaction (adults: *p* < 0.001, $\hat{\xi }$ = 0.53; adolescents: *p* < 0.001, $\hat{\xi }$ = 0.63).

#### Positive affect

An inspection of Fig. 3a and b indicated that positive affect was lowest for the Emotional-Unreliable network and highest for the Creative-Reliable network. The robust post-hoc analyses presented in Table 7 generally supported this observation. However, in adults it was questionable whether the Emotional-Unreliable network differed significantly in positive affect from the Organized-Reliable network ($\hat{\varphi }$ = −0.13, 95% CI [−0.29, 0.02], *p* = 0.037).

**Table 7:** Summary of robust post-hoc comparisons between temperament-character networks.

Variable	Comparison					
	network		Network	$\hat{\varphi }$	CI	*p*
Panel A: adult sample						
Positive Affect	Emotional-unreliable	–	Organized-reliable	− 0.13	[− 0.29, 0.02]	0.037
		**–**	Creative-reliable	− 0.47	[− 0.63, − 0.30]	< 0.001
	Organized-reliable	**–**	Creative-reliable	− 0.33	[− 0.51, − 0.16]	< 0.001
Negative affect	Emotional-Unreliable	**–**	Organized-reliable	0.63	[0.49, 0.77]	< 0.001
		**–**	Creative-reliable	0.74	[0.60, 0.89]	< 0.001
	Organized-reliable	**–**	Creative-reliable	0.11	[− 0.02, 0.25]	0.047
Life satisfaction	Emotional-Unreliable	**–**	Organized-reliable	− 0.34	[− 0.43, − 0.26]	< 0.001
		**–**	Creative-Reliable	− 0.38	[− 0.49, − 0.26]	< 0.001
	Organized-reliable	**–**	Creative-reliable	− 0.03	[− 0.15, 0.09]	0.526
Panel B: adolescent sample						
Positive affect	Emotional-unreliable	**–**	Organized-reliable	− 0.40	[− 0.51, − 0.28]	< 0.001
		**–**	Creative-reliable	− 0.63	[− 0.74, − 0.52]	< 0.001
	Organized-reliable	**–**	Creative-reliable	− 0.23	[− 0.36, 0.11]	< 0.001
Negative affect	Emotional-unreliable	**–**	Organized-reliable	0.84	[0.74, 0.95]	< 0.001
		**–**	Creative-reliable	0.61	[0.50, 0.72]	< 0.001
	Organized-reliable	**–**	Creative-reliable	− 0.23	[− 0.33, − 0.13]	< 0.001
Life satisfaction	Emotional-unreliable	**–**	Organized-reliable	− 1.03	[− 1.17, − 0.89]	< 0.001
		**–**	Creative-Reliable	− 1.06	[− 1.19, − 0.92]	< 0.001
	Organized-reliable	**–**	Creative-reliable	− 0.03	[− 0.14, 0.09]	0.596

#### Negative affect

From Fig. 3c and d it was evident that the Emotional-Unreliable network was associated with higher than average negative affect, while the Organized-Reliable and Creative-Reliable networks were associated with lower than average negative affect. Supporting this, post-hoc tests displayed in Table 7 showed the Emotional-Unreliable network differed significantly from the other two networks. In adults, it was questionable whether the Organized-Reliable network differed significantly in positive affect from the Creative-Reliable network ($\hat{\varphi }$ = 0.11, 95% CI [−0.02, 0.25], *p* = 0.047). However, in adolescents the paired-comparison showed the Creative-Reliable network had higher negative affect than the Organized-Reliable network ($\hat{\varphi }$ = −0.23, 95% CI [−0.33, −0.13], *p* < 0.001).

#### Life satisfaction

Figure 3e and f appeared to show the reverse pattern of Fig. 3c and d, with the Emotional-Unreliable network associated with lower life satisfaction than the Organized-Reliable and Creative-Reliable networks. In both samples, post-hoc comparisons confirmed these differences. Additionally, post-hoc analyses (Table 7) indicated that the Organized-Reliable and Creative-Reliable networks did not differ significantly from one another.

## Discussion

In this study, we explored the associations between the three dimensions of SWB–positive affect, negative affect, and life satisfaction–and components of personality that are shown by rigorous research to be regulated by genetically, functionally, and developmentally distinct psychobiological processes^22–25,34,45^. Adopting a similar procedure to past studies^47,62^ we tested how personality at various levels of descriptive complexity relates to SWB. Specifically, this involved testing how the three SWB dimensions relate to (a) individual temperament and character traits, (b) multi-trait temperament profiles and multi-trait character profiles, and (c) integrated temperament-character networks.

Using an analytical approach from prior works^26^ that allowed us to evaluate associations of individual temperament and character traits, we found that positive affect seemed to be largely dependent on persistence, self-directedness and self-transcendence. This finding aligns with others works that have demonstrated how human flourishing occurs when habits are persistently regulated to be in congruence with self-transcendent goals and values, thus resulting in a well-integrated personality^62^. Other constructs related to motivational persistence and perseverance, such as *grit*, have also been consistently associated with positive emotional experiences^84^.

Supporting current understanding that negative affect is not simply the absence of positive affect; we found that negative affect was linked to different personality traits. Specifically, negative affect was positively associated with high harm avoidance (which has specific subscales for fearfulness and shyness) and low self-directedness. This finding aligns with past works that have consistently shown how harm avoidance and low self-directedness are indicators of neuroticism and frequently associated with psychopathology and emotional/behavioral problems^85–89^. We also found that participants high in novelty seeking (and particularly adolescents) reported higher negative affect. Indeed, clinical research that has demonstrated that high novelty seeking is a precursor to emotional and behavioral problems^87,90^, such as substance abuse^91^, and Cluster B personality disorders^89^. The fact that negative affect was more strongly influenced by novelty seeking in adolescents than adults may reflect the relevance of this trait for the developmental process of identity formation and emancipation from adult authority, and immature capacity to self-regulate emotional impulses by character^92^. Within the sample of adolescents, one possibility is that unregulated novelty seeking, reflecting an impulsive eagerness to explore and try new things, may lead to more friction with adult authority and frustration at not being fully autonomous, and thus prompt negative affect.

As with negative affect, we found that life satisfaction was associated with harm avoidance and self-directedness (although with the opposite pattern of association). However, life satisfaction did not appear to be dependent on novelty seeking, instead revealing a trend for association with reward dependence in adolescents. Like novelty seeking, research shows reward dependence peaks in mid-adolescence, reflecting a high desire for peer approval^92^. The fact that satisfaction with life was more strongly associated reward dependence in adolescents than adults may again be because of their immature capacity to self-regulate emotional impulses by their own character, which results in a greater need for social support^92^. Within adolescents, one possibility is that reward dependence promotes higher satisfaction with life, particularly regarding friends and school, because it allows individuals to be more receptive to the norms and expectations of their peers^92^.

Robust evidence indicates that genes code for distinct temperament profiles rather than individual temperament traits^22,45^. In this person-centered study, we grouped participants into all theoretically possible configurations of the four temperaments. A first finding was that these temperament profiles were associated with differences in SWB. For example, participants with an explosive temperament (NHrp) typically reported lower than average positive affect and life satisfaction, and higher than average negative affect, suggesting that this temperament configuration is the least adaptive in terms of SWB. In contrast, adults and adolescents with a reliable temperament (nhRP) tended to report higher than average positive affect and life satisfaction, and lower than average negative affect. These findings are consistent with an emerging body of work that shows a reliable temperament is particularly adaptive for human functioning, with positive associations with student engagement^55^ and an overall ‘good character’^62^.

We also grouped participants into all theoretically possible character profiles. As predicted, the study indicated that SWB was strongly related to overall character development, with the ‘creative’ character (SCT) associated with the highest positive affect, lowest negative affect, and most life satisfaction. This finding aligns fully with evidence that the synergistic development of the three character traits allows an individual to cultivate the healthy practices of letting go (self-directedness), working in the service of others (cooperativeness), and growing in awareness of what is beyond the individual self (self-transcendence)^64,93^, which are key practices of virtue in action that promote SWB^26,28^. Indeed, this is why “third-wave psychotherapies” that aim to promote self-transcendence alongside self-directedness and cooperativeness (e.g., through mindfulness training), are more effective than more narrow cognitive-behavioral approaches^94,95^.

A major contribution of the study was that we explored, for the first time, how the three components of SWB differed between the personality networks that reflect differences in three major systems of human learning and memory^25^. Our results demonstrated that the capacity for self-control of emotional conflicts and goals, as is characteristic of people in the organized-reliable and creative-reliable networks, was beneficial for reducing negative affect and elevating hopeful cognitive appraisals about one’s circumstances. However, we found that only the combination of intentional self-control and self-awareness was associated with elevations in positive affect. This finding extends and clarifies earlier work showing that the emergence of self-awareness in behaviorally modern *Homo sapiens* around 100,000 years ago provided human beings the capacity for healthy longevity, creativity, and prosocial values and behavior^24^.

Our current findings align with previous conceptualizations of positive emotions as tools selected for their adaptive functions of broadening one’s horizon, leading to more flexible creative responses and the building of personal psychological and social resources^96^. Indeed, it has been demonstrated that the emergence of positive emotions has cascade effects into socio-cognitive processes such as attention ^97^, interpersonal cognition ^98^, logical reasoning ^99^ and creativity ^100^. In turn, while positive emotions energize people to explore and to think outside of the box, intentional self-control and self-awareness provide the flexibility needed for growth, and innovative problem resolution, as well as the structure needed for effective, management of the available resources. Therefore, based on our findings, as well as the latest genetic, neurobehavioral, and evolutionary evidence presented throughout this study, we argue that the psychobiological model offers an explanatory holistic framework that is capable of adequately addressing the prominent non-linear and dynamic links between emotion and cognition, which promote positive human functioning and flourishing.

A major implication of the study results for clinical practice is that they add to a growing recognition that an understanding of a whole person’s adaptive functioning and well-being requires an integration of interdependent brain networks for emotional reactivity, rational self-government, and self-awareness. Put differently, the study highlights that the path to a happy and healthy life depends not on one key feature, but rather the integration of the physical (biological), mental, and spiritual aspects of human functioning^101^. Therefore, clinical practitioners need to adopt person-centered interventions that aim to cultivate growth of the whole person^64^. Additionally, the study shows that higher positive affect is dependent on different psychobiological processes than life satisfaction and negative affect, namely those associated with the capacity for self-awareness such as insight and creativity. As such, the cultivation of human flourishing via positive emotionality specifically requires the growing in awareness of oneself, via reflective and meditative practices, as an entangled aspect of something greater than one’s individual self (that is, extending to human communities, nature, the universe, and possibly what is sacred and divine^64^).

Despite the growing number of empirical studies on SWB–including recently on the association between psychobiological personality dimensions and SWB^102^–there remain various research questions that require further investigation. Firstly, most research on this topic has used cross-sectional designs. However, research has demonstrated that temperament and character dimensions develop across the lifespan^51,92^, and therefore it is important to explore with longitudinal designs how intraindividual change in personality over time relates to changes in SWB. Second, while studies strongly support the association between personality and SWB dimensions, it remains necessary to explicate how these associations interact with societal, environmental, and cultural variables. A reasonable hypothesis, for example, might be that factors like income vary in their association with SWB as a function of personality. As a related issue, it is important to conduct empirical studies to develop understanding of how human personality relates to outcomes such as health and longevity via its association with SWB.

It is important to note limitations that may constrain the generality of our findings^103^. We acknowledge that specific characteristics of the study samples (e.g., adolescent sample comprising only 9th graders) may limit generalizability of our findings. We also note our exclusive use of self-report instruments, although argue that the TCI-R and JTCI have been validated extensively, and therefore expect our results to be reproducible. Finally, we draw attention to the fact that data collection for the adolescent sample occurred during the COVID19 pandemic, meaning we cannot fully discount whether the same findings would be found outside of the pandemic.

## Conclusions

The study findings indicate that individual differences in positive affect, negative affect and life satisfaction are dependent on distinct organizations of psychobiological systems and processes. Most notably, we found that the organization of the relations among temperament and character profiles, captured by more-or-less integrated personality networks that take into account the intentional and creative self-regulatory processes within individuals was strongly related to all aspects of SWB. Specifically, negative affect and life satisfaction were dependent on a personality network for intentional self-control, while positive affect was uniquely dependent on the personality network for self-awareness. This finding empirically demonstrates, for the first time, an association between positive affect and a genetically distinct personality network linked to the unique human capacities for creativity, healthy longevity, prosocial behavior, and self-awareness.

### Supplementary Information

Supplementary Information.

## Data Availability

The datasets generated during and/or analyzed during the current study are not publicly available because the TCI-R is copyrighted and associated data is proprietary. Non TCI-R data are however available from the Corresponding author upon reasonable request.
