# Author Correction: Disentangling the personality pathways to well-being

**DOI:** 10.1038/s41598-023-45105-3

**Published:** 2023-10-19

**Authors:** Paulo A. S. Moreira, Richard A. Inman, C. Robert Cloninger

**Affiliations:** 1 Instituto de Psicologia E de Ciências da Educação (IPCE), Universidade Lusíada Porto, Porto, Portugal; 2 Centro de Investigação Em Psicologia Para O Desenvolvimento (CIPD), Lisbon, Portugal; 3 Department of Psychiatry, Washington University in St. Louis, St. Louis, MO USA; 4 Anthropedia Foundation, St. Louis, USA; 5 Departamento de Educação e Psicologia, Faculdade de Ciências Humanas e Sociais, University of Trás-Os-Montes and Alto Douro (UTAD), Quinta de Prados, 5000-801 Vila Real, Portugal

Correction to: *Scientific Reports* 10.1038/s41598-023-29642-5, published online 27 February 2023

The original version of this Article contained an error in the Acknowledgments section.

“This work was supported in part by funding from the Fundação para a Ciência e Tecnologia (FCT) [grant number: PTDC/CED-EDG/31615/2017].”

now reads:

“This work was supported by national funds, from the Fundação para a Ciência e Tecnologia (FCT), in the scope of the project UIDB/04375/2020.”

The original Article has been corrected.

