# Supplementary material for: Disentangling the personality pathways to well-being
Source: Sci Rep. 2023 Feb 27;13:3353. doi: 10.1038/s41598-023-29642-5 (PMC9969391; doi:10.1038/s41598-023-29642-5)
Supplement: Supplementary file 1 — Supplementary Information. [file 41598_2023_29642_MOESM1_ESM.docx]

**Supplementary Materials**

**Supplementary Table 1.** Variable descriptive statistics (adults)

**Supplementary Table 2.** Variable descriptive statistics (adolescents)

**Supplementary Table 3.** Gender distributions and average ages of participants grouped in each temperament profile, character profile, and temperament-character network

**Supplementary Table 4.** Frequency table showing distribution of temperament and character profiles within the temperament-character networks (Adults).

**Supplementary Table 5.** Frequency table showing distribution of temperament and character profiles within the temperament-character networks (Adolescents).

| **Supplementary Table 1.** Variable descriptive statistics (adults) | | | | |
| --- | --- | --- | --- | --- |
|  | Mean | SD | Skew | Kurtosis |
| Subjective Well-being |  |  |  |  |
| Positive Affect | 3.23 | 0.69 | -0.39 | 0.21 |
| Negative Affect | 1.93 | 0.70 | 0.91 | 0.44 |
| Life Satisfaction | 3.84 | 0.45 | -0.41 | 0.69 |
| Temperament |  |  |  |  |
| Novelty Seeking | 96.09 | 10.81 | 0.27 | 0.97 |
| Harm Avoidance | 99.68 | 15.70 | 0.35 | 0.63 |
| Reward Dependence | 99.11 | 11.65 | -0.17 | 0.80 |
| Persistence | 122.13 | 14.30 | 0.05 | 0.55 |
| Character |  |  |  |  |
| Self-Directedness | 138.61 | 16.03 | -0.19 | 0.19 |
| Cooperativeness | 134.78 | 13.31 | -0.43 | 0.86 |
| Self-Transcendence | 79.57 | 12.79 | 0.04 | 0.11 |

| **Supplementary Table 2.** Variable descriptive statistics (adolescents) | | | | |
| --- | --- | --- | --- | --- |
|  | Mean | SD | Skew | Kurtosis |
| Subjective Well-being |  |  |  |  |
| Positive Affect | 3.01 | 0.79 | -0.15 | -0.16 |
| Negative Affect | 2.01 | 0.79 | 0.91 | 0.67 |
| Life Satisfaction | 4.61 | 1.01 | -0.83 | 0.60 |
| Temperament |  |  |  |  |
| Novelty Seeking | 62.64 | 10.61 | 0.08 | -0.12 |
| Harm Avoidance | 56.04 | 9.09 | 0.04 | -0.02 |
| Reward Dependence | 51.04 | 7.31 | -0.10 | 0.01 |
| Persistence | 61.98 | 9.64 | 0.00 | -0.17 |
| Character |  |  |  |  |
| Self-Directedness | 87.06 | 11.29 | 0.10 | -0.56 |
| Cooperativeness | 75.12 | 10.56 | -0.45 | -0.44 |
| Self-Transcendence | 32.65 | 5.49 | -0.16 | 0.07 |

| **Supplementary Table 3.** Gender distributions within, and average ages of, each temperament profile, character profile, and temperament-character network. Values given for age are means ± SD. | | | | | | |
| --- | --- | --- | --- | --- | --- | --- |
|  | Adults | | | Adolescents | | |
|  | N | % Male | Age, years | N | % Male | Age, years |
| Full Sample | 897 | 30 | 35.7 ± 16.8 | 1,739 | 43 | 14.2 ± 0.72 |
| **Temperament Profile** | Gender: *p* < .001  Age: *p* < .001,$\hat{\xi}$ = .39 | | | Gender: *p* < .001  Age: *p* < .001,$\hat{\xi}$ = .38 | | |
| nhrp “Independent” | 19 | 53 | 46.6 ± 17.2 | 68 | 76 | 14.4 ± 0.83 |
| nhrP | 50 | 42 | 39.0 ± 16.1 | 100 | 64 | 14.1 ± 0.66 |
| nhRp “Reliable” | 15 | 47 | 38.7 ± 17.1 | 17 | 71 | 14.2 ± 0.97 |
| nhRP | 40 | 33 | 38.0 ± 16.7 | 213 | 37 | 14.1 ± 0.58 |
| nHrp “Methodical” | 85 | 28 | 39.6 ± 19.5 | 109 | 49 | 14.4 ± 0.84 |
| nHrP | 59 | 31 | 36.4 ± 17.9 | 100 | 30 | 14.2 ± 0.67 |
| nHRp “Cautious” | 28 | 25 | 38.4 ± 20.0 | 67 | 31 | 14.4 ± 0.85 |
| nHRP | 37 | 14 | 28.3 ± 13.7 | 252 | 19 | 14.0 ± 0.46 |
| Nhrp “Adventurous” | 40 | 53 | 38.3 ± 15.5 | 81 | 80 | 14.5 ± 1.01 |
| NhrP | 46 | 48 | 31.0 ± 13.7 | 32 | 63 | 14.1 ± 0.44 |
| NhRp “Passionate” | 25 | 32 | 32.5 ± 14.4 | 15 | 47 | 14.3 ± 0.72 |
| NhRP | 61 | 33 | 28.9 ± 12.0 | 38 | 37 | 13.9 ± 0.53 |
| NHrp “Explosive” | 78 | 27 | 36.4 ± 16.8 | 178 | 60 | 14.4 ± 0.92 |
| NHrP | 29 | 17 | 34.5 ± 17.0 | 33 | 27 | 14.0 ± 0.39 |
| NHRp “Sensitive” | 44 | 14 | 27.3 ± 14.1 | 62 | 27 | 14.1 ± 0.56 |
| NHRP | 43 | 14 | 27.9 ± 12.4 | 50 | 20 | 14.1 ± 0.51 |
| **Character Profile** | Gender: *p* = .038  Age: *p* < .001,$\hat{\xi}$ = .35 | | | Gender: *p* < .001  Age: *p* < .001,$\hat{\xi}$ = .22 | | |
| Sct “Apathetic” | 116 | 40 | 34.9 ± 15.8 | 341 | 62 | 14.3 ± 0.85 |
| scT “Disorganized” | 123 | 34 | 36.7 ± 19.6 | 139 | 55 | 14.4 ± 0.93 |
| sCt “Dependent” | 39 | 31 | 26.2 ± 12.2 | 78 | 27 | 14.3 ± 0.80 |
| sCT “Moody” | 77 | 16 | 28.1 ± 14.3 | 185 | 19 | 14.2 ± 0.61 |
| Sct “Bossy” | 65 | 28 | 41.8 ± 16.1 | 88 | 69 | 14.1 ± 0.55 |
| ScT “Fanatical” | 33 | 24 | 36.6 ± 16.9 | 61 | 66 | 14.2 ± 0.70 |
| SCt “Organized” | 112 | 28 | 35.9 ± 16.7 | 292 | 40 | 14.1 ± 0.54 |
| SCT “Creative” | 117 | 32 | 36.1 ± 16.1 | 407 | 30 | 14.1 ± 0.70 |
| **Network** | Gender: *p* = .509  Age: *p* < .001,$\hat{\xi}$ = .17 | | | Gender: *p* < .001  Age: *p* < .001,$\hat{\xi}$ = .18 | | |
| Emotional-Unreliable | 355 | 32 | 33.1 ± 17.0 | 743 | 47 | 14.3 ± 0.81 |
| Organized-Reliable | 210 | 27 | 38.3 ± 16.7 | 441 | 49 | 14.1 ± 0.56 |
| Creative-Reliable | 117 | 32 | 36.1 ± 16.1 | 407 | 30 | 14.1 ± 0.70 |

| **Supplementary Table 4.** Frequency table showing distribution of temperament and character profiles within the temperament-character networks (Adults) | | | | | | | | | | | | | | | | | | | |
| --- | --- | --- | --- | --- | --- | --- | --- | --- | --- | --- | --- | --- | --- | --- | --- | --- | --- | --- | --- |
|  |  | nhrp | nhrP | nhRp | nhRP | nHrp | nHrP | nHRp | nHRP | Nhrp | NhrP | NhRp | NhRP | NHrp | NHrP | NHRp | NHRP |  |  |
| Emotional-Unreliable | sct | 1 | 3 | 0 | 0 | 19 | 7 | 4 | 1 | 12 | 4 | 3 | 0 | 33 | 5 | 9 | 2 | 103 | 289 |
|  | scT | 0 | 7 | 1 | 0 | 12 | 13 | 2 | 1 | 3 | 8 | 4 | 6 | 16 | 10 | 5 | 6 | 94 |  |
|  | sCt | 1 | 0 | 1 | 0 | 9 | 2 | 3 | 0 | 1 | 2 | 1 | 2 | 3 | 0 | 6 | 3 | 34 |  |
|  | sCT | 0 | 1 | 1 | 5 | 6 | 5 | 3 | 5 | 0 | 0 | 2 | 8 | 1 | 1 | 11 | 9 | 58 |  |
| Organized-Reliable | Sct | 1 | 9 | 1 | 3 | 5 | 0 | 0 | 2 | 7 | 7 | 2 | 2 | 2 | 4 | 2 | 1 | 48 | 156 |
|  | ScT | 0 | 3 | 1 | 1 | 0 | 3 | 0 | 1 | 2 | 6 | 1 | 4 | 0 | 1 | 0 | 2 | 25 |  |
|  | SCt | 7 | 5 | 7 | 5 | 10 | 8 | 2 | 5 | 4 | 3 | 3 | 15 | 2 | 0 | 4 | 3 | 83 |  |
| Creative-Reliable | SCT | 3 | 9 | 3 | 19 | 2 | 10 | 4 | 9 | 4 | 4 | 9 | 16 | 1 | 0 | 0 | 4 | 97 | 97 |
|  |  | 13 | 37 | 15 | 33 | 63 | 48 | 18 | 24 | 33 | 34 | 25 | 53 | 58 | 21 | 37 | 30 | 542 |  |

| **Supplementary Table 5.** Frequency table showing distribution of temperament and character profiles within the temperament-character networks (Adolescents) | | | | | | | | | | | | | | | | | | | |
| --- | --- | --- | --- | --- | --- | --- | --- | --- | --- | --- | --- | --- | --- | --- | --- | --- | --- | --- | --- |
|  |  | nhrp | nhrP | nhRp | nhRP | nHrp | nHrP | nHRp | nHRP | Nhrp | NhrP | NhRp | NhRP | NHrp | NHrP | NHRp | NHRP |  |  |
| Emotional-Unreliable | sct | 22 | 11 | 2 | 0 | 42 | 8 | 3 | 4 | 44 | 6 | 2 | 1 | 98 | 9 | 9 | 3 | 264 | 590 |
|  | scT | 2 | 1 | 0 | 1 | 11 | 6 | 4 | 0 | 10 | 6 | 1 | 2 | 49 | 8 | 10 | 1 | 112 |  |
|  | sCt | 2 | 2 | 0 | 2 | 11 | 6 | 11 | 17 | 1 | 0 | 1 | 0 | 6 | 1 | 2 | 3 | 65 |  |
|  | sCT | 1 | 3 | 0 | 2 | 22 | 24 | 16 | 33 | 0 | 2 | 2 | 2 | 11 | 4 | 19 | 8 | 149 |  |
| Organized-Reliable | Sct | 14 | 9 | 3 | 1 | 3 | 3 | 1 | 7 | 10 | 1 | 1 | 4 | 1 | 2 | 0 | 3 | 63 | 369 |
|  | ScT | 4 | 5 | 0 | 4 | 3 | 8 | 3 | 3 | 6 | 5 | 2 | 2 | 2 | 0 | 3 | 2 | 52 |  |
|  | SCt | 5 | 35 | 6 | 106 | 5 | 12 | 5 | 54 | 1 | 4 | 3 | 6 | 0 | 1 | 3 | 6 | 252 |  |
| Creative-Reliable | SCT | 8 | 25 | 3 | 89 | 3 | 24 | 15 | 120 | 4 | 1 | 1 | 19 | 3 | 2 | 9 | 21 | 347 | 347 |
|  |  | 58 | 91 | 14 | 205 | 100 | 91 | 58 | 238 | 76 | 25 | 13 | 36 | 170 | 27 | 55 | 47 | 1304 |  |


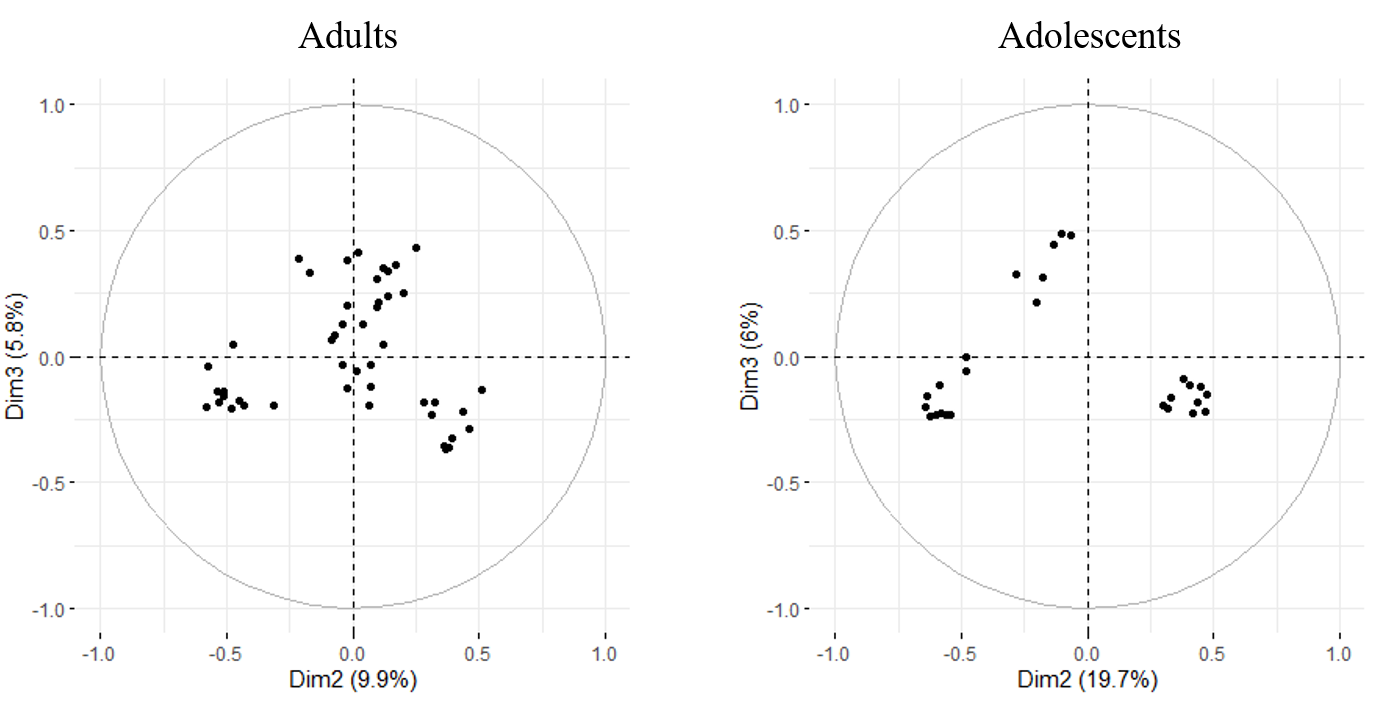


**Supplementary Figure 1.** Variable correlation plots showing the relationship between SWB items relative to the second and third principal components (excluding the first component reflective of a general SWB factor).
